# Distraction descriptor for brainprint authentication modelling using probability-based Incremental Fuzzy-Rough Nearest Neighbour

**DOI:** 10.1186/s40708-023-00200-z

**Published:** 2023-08-05

**Authors:** Siaw-Hong Liew, Yun-Huoy Choo, Yin Fen Low, Fadilla ‘Atyka Nor Rashid

**Affiliations:** 1 Faculty of Computer Science and Information Technology, Universiti Malaysia Sarawak (UNIMAS), 94300 Kota Samarahan, Sarawak Malaysia; 2 Faculty of Information and Communication Technology, Universiti Teknikal Malaysia Melaka (UTeM), 76100 Durian Tunggal, Melaka, Malaysia; 3 Faculty of Electronics and Computer Engineering, Universiti Teknikal Malaysia Melaka (UTeM), 76100 Durian Tunggal, Melaka, Malaysia; 4 Faculty of Information Science and Technology, Universiti Kebangsaan Malaysia (UKM), 43600 Bangi, Selangor Malaysia

**Keywords:** Distraction descriptor, Probability-based IncFRNN, Brainprint authentication, Object variation

## Abstract

This paper aims to design distraction descriptor, elicited through the object variation, to refine the granular knowledge incrementally, using the proposed probability-based incremental update strategy in Incremental Fuzzy-Rough Nearest Neighbour (IncFRNN) technique. Most of the brainprint authentication models were tested in well-controlled environments to minimize the influence of ambient disturbance on the EEG signals. These settings significantly contradict the real-world situations. Thus, making use of the distraction is wiser than eliminating it. The proposed probability-based incremental update strategy is benchmarked with the ground truth (actual class) incremental update strategy. Besides, the proposed technique is also benchmarked with First-In-First-Out (FIFO) incremental update strategy in K-Nearest Neighbour (KNN). The experimental results have shown equivalence discriminatory performance in both high distraction and quiet conditions. This has proven that the proposed distraction descriptor is able to utilize the unique EEG response towards ambient distraction to complement person authentication modelling in uncontrolled environment. The proposed probability-based IncFRNN technique has significantly outperformed the KNN technique for both with and without defining the window size threshold. Nevertheless, its performance is slightly worse than the actual class incremental update strategy since the ground truth represents the gold standard. In overall, this study demonstrated a more practical brainprint authentication model with the proposed distraction descriptor and the probability-based incremental update strategy. However, the EEG distraction descriptor may vary due to intersession variability. Future research may focus on the intersession variability to enhance the robustness of the brainprint authentication model.

## Introduction

Brain signals are being studied within the medical field for brain disorders such as Alzheimer, schizophrenia, spinal cord injuries, epilepsy, and stroke among the others. Furthermore, they are also applied in assistive, rehabilitative, and entertainment applications as the basis for Brain–Computer Interface (BCI) and Brain–Machine Interface (BMI). Despite widespread interest in clinical applications, the utilization of brain signals such as Electroencephalogram (EEG) is used as a biometric modality for person authentication or person identification [1–3]. EEG signal is an outstanding biometric modality with several benefits. The EEG signal would be more difficult to steal, and it would be cancellable. Numerous unique brain rhythms can trigger the EEG signals. For instance, when the stored brainprint of a client is stolen, disclosed, or lost, a different brainprint can be generated from a specific type of brain activities and responses. For example, the EEG signals can be recorded from different colour stimuli [4] or black and white stimuli [5]. Thus, a new brainprint could be used to substitute the stolen one. Furthermore, EEG is an example of biodynamic signal, demonstrating the evidence of personal aliveness. EEG is also proven to have low intra-subject and high inter-subject variability [6]. Thus, brainprint authentication research has progressed rapidly [7–9], in conjunction with the growth of portable low cost but high time resolution acquisition devices over the past few years [10]. EEG signals are usually nonlinear, nonstationary, and difficult to recreate due to the impact of several noise sources such as environmental noise and physiological noise [5, 11]. Current research normally conducted the EEG recording in a very quiet environment to minimize the disturbance [12, 13]. However, these environments are highly artificial and significantly differ from real-world situations, where people have to handle the environmental distractions. Thus, making use of the distraction is wiser than eliminating it. Human response to physiological noises is unique, but no concrete studies utilize distraction represented in EEG signals as biometric descriptors complementary to current biometric features.

A practical brainprint authentication model should always encapsulate the changes and variations of the EEG signals. Incremental learning has its capability to gradually remodel and reform the current knowledge granules incrementally for the purpose of detecting the new changes in the EEG signals. The commonly used incremental update strategies are the actual class and First-In-First-Out (FIFO) update strategies. The actual class label represents the ground truth. However, it is almost impossible to obtain the actual class label in brainprint authentication applications. In contrast, the FIFO incremental update strategy stores the new test objects and eliminates the oldest objects without considering the actual class label. It cannot ensure that only the nonrepresentative EEG signals will be eliminated, especially for the imbalanced class distribution dataset. Thus, it is crucial to modify the current incremental update strategy to implement in real-world situations. The proposed probability-based Incremental Fuzzy-Rough Nearest Neighbour (IncFRNN) is a modified version from IncFRNN [14] by introducing the probability method to overcome the use of actual class in the incremental update strategy. The proposed probability-based incremental update strategy imposes the variation of an object, object insertion, and object deletion. The main idea of the object insertion is to capture the new changes of the individual features from the EEG data due to the EEG is a nonstationary signal that fluctuates over the time.

The rest of this paper is structured as follows: Sect. 2 is a literature review on the brain responses towards environmental distractions and the existing incremental update strategies. Section 3 presents the existing IncFRNN algorithm and the proposed probability-based IncFRNN algorithm, respectively. Section 4 outlines the materials and methods, which includes the data acquisition, EEG distraction descriptor, feature selection, classification, and performance measurement and validation test. Section 5 depicts the experimental results and Section 6 provides the discussion on the findings. Section 7 draws the conclusion and suggests the direction of future work.

## Literature review

Research on using brainprint authentication is catching attention in recent years. However, there are many challenges must be resolved before considering its application in real-life circumstances. EEG signals are susceptible to any environmental disturbance due to the weaknesses of the signals. Thus, most of the research on EEG signals recording is generally conducted in a very quiet room to minimize the disturbance. By doing this way, it is able to acquire the best quality of the EEG signals. However, one of the challenges is the EEG signals acquisition process in the current controlled environment is highly artificial and significantly differs from real-world situations. It is a critical issue but the research to address this problem is still limited [15]. Besides that, emotion is also another essential issue need to be addressed, and so far, there is lack of research to tackle this challenge. A complex psychological state known as emotion, which involves three unique components: a physiological response, a behavioural response, and a subjective experience [16]. Four critical points such as the environment and equipment setting, emotion elicitation procedure, evaluation of categories of stimuli, and evaluation of individual differences should take into account to ensure the recorded EEG signals are related to the emotion rather than physical features [17]. One way to close this gap is to simulate different environments during EEG signals acquisition to investigate the effects of distraction towards the subjects. Instead of minimizing the disturbance, it is potential to use as a distraction descriptor to complement the current brainprint authentication model. It is because the EEG signals are well known to be unique across individual as every individual think and response differently.

Alpha band is frequently used to describe individual differences as a trait variable [18]. Large inter-individual differences in alpha frequency are correlated with the age and memory performance [19]. The mean of alpha frequency increases with age from childhood to puberty. The alpha frequency begins to decline when increasing age after puberty [20]. The inter-subject variability is a large degree explained by genetic factors, even the research on the heritability of twins is estimated at 80% [21]. Different individuals have alpha waves with different wavelengths. The processing of information is accelerated by higher alpha frequency. Thus, there are correlations between the inter-subject differences in alpha peak frequency and several cognitive measures, as well as information processing and reaction times. A good performer retrieves the information from memory faster than a lousy performer. Hence, it is crucial to note that these findings are based on the inter-individual variability of alpha frequency, which was found to be significantly related to the inter-individual differences in the speed information processing.

Other than that, beta band oscillations are the oscillations in the human brain in the frequency range of 13–30 Hz. The beta band indicates the variations in sensorimotor behaviour accompanied by different types of attention, which are endogenous and exogenous [22]. Beta band activity was proven served as a carrier for attentional activation in cortical centres of the visual system [23]. An increase of beta band was found during the anticipatory period. The authors showed that faster responses to target stimuli increased the alertness with higher EEG activation in the beta band over the occipito-parietal regions [24]. Gola et al. [22] proved that the beta band recorded over the occipital areas is related to visual attention.

With the use of incremental learning model, a system is able to learn from the new information when it is available. It does not required to reconstruct or retrain the machine learning models from the scratch. In the perspective of machine learning, a good training set should have all the knowledge to achieve the best system performance. Nowadays, the data volume grows rapidly and changes over time. There is an increasing amount of accumulated data being unprocessed because the batch leaning is unable to handle the large number of data in the given period. On the other hand, incremental learning trains the model wisely to conquer the drawbacks of batch learning. The incremental learning capability is getting more important for machine learning applications along with the growth of the data volume. Incremental learning is an effective dynamic data mining technology that can gain information from the current data more quickly based on the prior knowledge from previous data and correlation of real-time data. Rather than synthesizing all of the information provided, the incremental algorithms attempt to avoid remembering useless information. This is very suitable to be used in real-world applications which enable the models adapt to the new changing environments. The cost of data storage and maintenance can be reduced due to the incremental learning allows the model to use the information whenever it is available, resulting in the models that are always up to date. It is incredibly essential since many real-time applications do not require sufficient training data because the learning can be a continuing process [25]. Thus, it is suitable for different applications such as model personalization, learning in changing environments, robotics, image processing, signal processing, automated annotation, outlier detection, data analytics, big data processing, and Internet of Things technology.

An information system or structured data comprise of three elements, which are the object (instance); the attribute (feature); and the attribute value. Thus, the incremental update strategies are the variation of an object; the variation of an attribute; and the variation of attribute values. The variation of an object includes the insertion and deletion of an object; the variation of an attribute includes the insertion and deletion of an attribute; and the variation of attribute values focuses on the coarsening and refining of attribute values. Among these incremental update strategies, the variation of object is widely used. A new object is inserted to the information system, and another object may remove from the information system. Meanwhile, the deletion of the object is optional, and it depends on the necessity. The main purpose of object insertion is to include the new or changing information and update the lower and upper approximations. The new knowledge is extracted and continuously updated the information system; otherwise, it could be outdated. The variation of an object includes the new object to update the information system over time, while the number of attributes remains the same.

In machine learning, instance-based learning has usually implemented the variation of an object to incrementally update the knowledge base. Instance-based learning is a supervised classification learning algorithm that operates after comparing current objects with the previously trained objects stored in the memory. The primary benefit of instance-based learning over the other machine learning techniques is the ability of model adaptability to the previously unseen data. Thus, the training set is always updated and will not degrade the performance. K-Nearest Neighbour (KNN), Fuzzy-Rough Nearest Neighbours (FRNN), Naïve Bayes, and Support Vector Machine (SVM) are the examples of instance-based learning techniques.

KNN is one of the popular instance-based learning classification techniques in pattern recognition, text mining, stock market prediction, and so forth [26]. This supervised machine learning algorithm is straightforward and it could be used to address both regression and classification tasks. In KNN algorithm, it finds the $k$ number of closest neighbours from the training objects and uses the majority vote of testing objects to assign the decision class. In recent years, KNN has been designed with incremental learning, which allows the information system updated continuously whenever the new objects arrived. KNN implements lazy learning approach, which stored all the available objects as training objects. It removes the oldest test objects from the training pool and stores the new one. In another words, KNN implements First-In-First-Out (FIFO) incremental update strategy to revise the knowledge granules incrementally. The newly inserted object will be stored in the training pool and used as the train object for the next incoming test object [27]. On the other hand, FRNN is a hybrid technique that merging the strength of fuzzy sets and the rough sets, and the nearest neighbours classification approach. FRNN is modified from the KNN algorithm that make use of fuzzy-rough set theory [28]. The FRNN determines the nearest neighbours by using the fuzzy similarity measure rather than the Euclidean distance. The notion of fuzzy-rough set was introduced to complement each counterpart with impreciseness and vagueness [29]. The fundamental definition of the fuzzy-rough set model is the fuzzy lower and upper approximations. The Incremental Fuzzy-Rough Nearest Neighbour (IncFRNN) algorithm [5] was introduced to solve the incremental learning problem in EEG signals analysis. The FRNN algorithm has been improved with the additional process layer for actual class incremental update and the window size control, which is called IncFRNN algorithm. The primary concept of the IncFRNN algorithm is to update the knowledge granules incrementally based on the actual class label through object insertion and object deletion processes. As a result, the knowledge granules are able to self-adapt towards the new changes environment by capturing new characteristic changes that represent the current individual biometric traits during the authentication process.

SVM is also frequently used in EEG signals classification. It aims to find the maximum-margin hyperplane in multi-dimensional space that distinctly classifies the feature vectors. However, it is more complicated as compared to the KNN. The choice of parameters and algorithmic complexity could have an impact on the training time especially when dealing with the large datasets. The complexity highly depends on the size of the dataset [30] and the traditional SVM is unable to adapt the online learning environment. Thus, the online SVM has to retrain the model with the current support vectors and new incoming data [31]. Han et al. [32] introduced a new incremental SVM to improve the efficiency in handling large scale data. With the use of hyperplane information, the new incremental SVM made the offset of the hyperplane as little as possible. The experimental results indicated that the proposed technique had outperformed than the traditional SVM in the classification task. However, the incremental SVM algorithm requires the actual class label for all the input training data [33]. Apart from that, incremental Naïve Bayes algorithm was proposed in [34] by integrating instance-based learning. The proposed incremental Naïve Bayes model starts with calculating the probabilities for each class during the classification of a test object and validated with 34 benchmark datasets. The experimental results have proven that the simplicity in using the probability-based incremental update approach works well for pattern classification. However, despite these findings, no universal approach will necessarily be successful.

## Probability-based update strategy in Incremental Fuzzy-Rough Nearest Neighbour

Probability-based Incremental Fuzzy-Rough Nearest Neighbour (IncFRNN) is modified from the IncFRNN proposed in [5]. The incremental update strategy focuses on the variation of an object, object insertion, and object deletion. Incremental learning based on the variation of an object is good to improve the classification performance through iterative knowledge updates. The information system is changed by inserting or deleting the object, thus eliminating the need to retrain the learning model from scratch [35]. Indirectly, it can save the memory usage and the time taken to retrain the model.

The IncFRNN algorithm proposed in [5] used actual class incremental update strategy to update the training pool. The new test object will be inserted to the training pool when it is classified incorrectly. It is because the current knowledge granules have its limitation in predicting the test object. This update will help the authentication process whenever the model comes across the similar test object in the future. The IncFRNN algorithm is shown in Algorithm 1.

**Figure:**
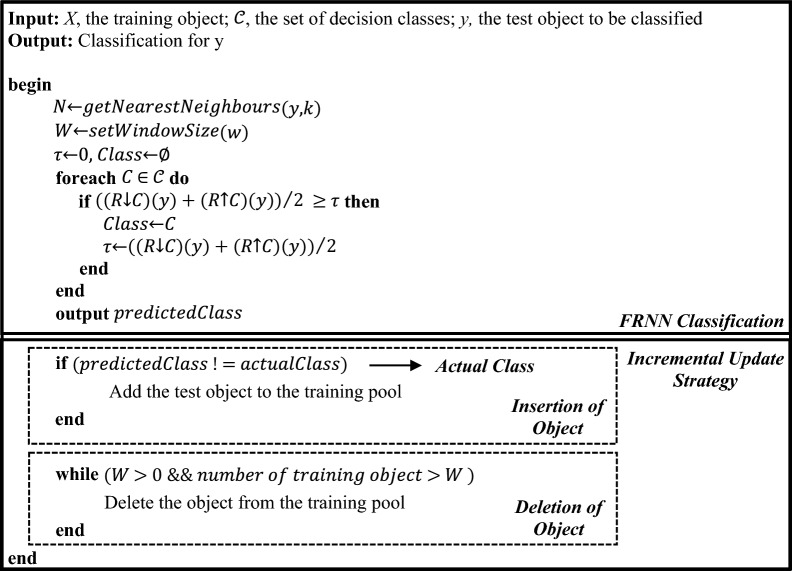
Algorithm 1: IncFRNN Algorithm [5]

The IncFRNN technique improves the capability of handling the nonstationary characteristics of EEG signals. However, it is not practical in real-world applications due to the mandatory requirement on actual class label to support the incremental updating strategy. It is almost impossible to obtain the actual class label in brainprint authentication applications. Due to the imbalanced class distribution in the EEG dataset, the First-In-First-Out (FIFO) incremental update strategy is less appropriate for brainprint authentication modelling. Thus, the probability-based incremental update strategy is proposed. The probability-based IncFRNN algorithm is shown in Algorithm 2.

**Figure:**
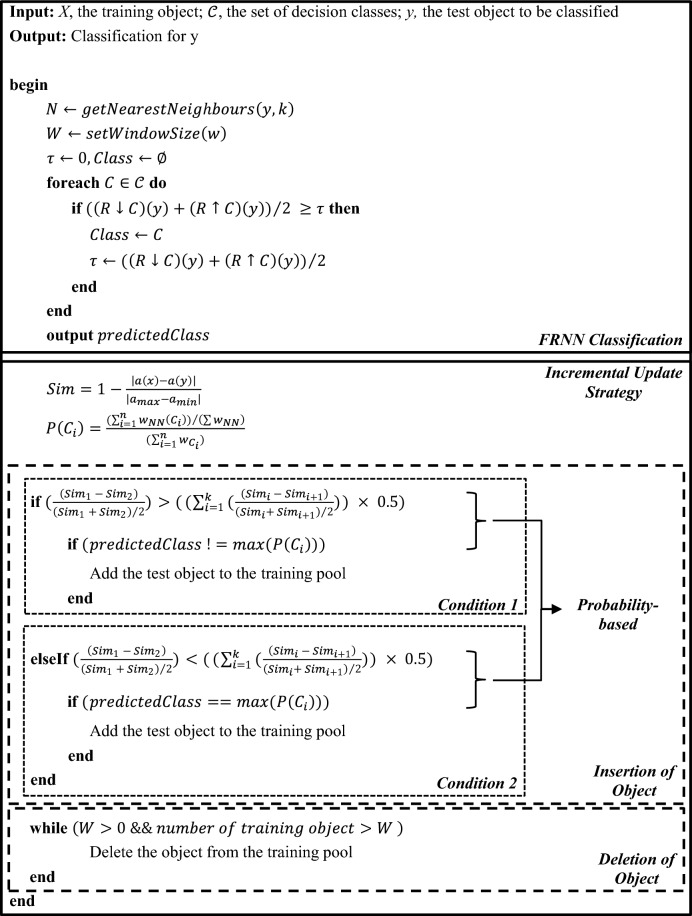
Algorithm 2: Probability-based IncFRNN algorithm

Based on Algorithm 1 and Algorithm 2, both IncFRNN and probability-based IncFRNN algorithms consist of two major parts, which are the classification and the incremental update strategy. The classification process in both the probability-based IncFRNN and the IncFRNN algorithms are based on the original FRNN algorithm, proposed by Jensen and Cornelis [36]. In the FRNN algorithm, the fuzzy tolerance relation, $R$, is shown as follows:

Given a set of conditional attributes ${\mathbb{A}}$, $R$ is defined by

1 $$R\left(x,y\right)= \underset{a\in {\mathbb{A}}}{\mathrm{min}}\left(1-\frac{\left|a\left(x\right)-a\left(y\right)\right|}{\left|{a}_{\mathrm{max}}-{a}_{\mathrm{min}}\right|}\right),$$

where $x$ is the object in attribute $a$, $y$ is the test object, and ${a}_{\mathrm{max}}$ and ${a}_{\mathrm{min}}$ are the maximal and minimal occurring value of attribute $a$.

Each decision class in the training data is iteratively examined by the FRNN classification algorithm. The lower and upper approximations for each class are taken into account when computing the membership of the testing data. When the values of $\left(R\downarrow C\right)\left(y\right)$ is high, it indicates that all the neighbours of $y$ are belonging to $C$. On the contrary, when the values of $\left(R\uparrow C\right)\left(y\right)$ is high, it indicates that at least one of the neighbours of $y$ is belonging to $C$.

The incremental learning process is carried out after obtaining the predicted class. The existing actual class incremental update strategy is shown in Algorithm 1, while the proposed probability-based incremental update strategy is shown in Algorithm 2. Probability-based incremental update strategy aims to include the new representative objects into the training pool without considering the actual class label of the test objects. The proposed probability-based incremental update strategy comprises three concepts from the literature review, which are the probability [37] measure, the intra and inter-subject variability [38], and the fuzzy similarity [36].

### Insertion of object

The main idea of inserting an object is to update the knowledge granules with the new objects when the current knowledge granules are unable to predict the new test object. There are two prerequisites to be considered: (a) the percentage of the difference between the top two nearest neighbours and (b) the predicted class of the test object and the class label with the highest probability. The formulas to calculate the threshold and the probability are illustrated in Eqs. (3) and (4), respectively.

In the probability-based IncFRNN algorithm, the threshold indicates the degree of difference in the nearest neighbours based on the similarity value. It calculates the total of difference in all the nearest neighbours. First, the similarity of the test object and training objects is computed by using Eq. (2):

2 $$\mathrm{Sim}=1-\frac{\left|a\left(x\right)-a\left(y\right)\right|}{\left|{a}_{\mathrm{max}}-{a}_{\mathrm{min}}\right|},$$

where $a\left(x\right)$ is the training object of attribute $a$, $a\left(y\right)$ is the test object, and ${a}_{\mathrm{max}}$ and ${a}_{\mathrm{min}}$ are the maximal and minimal occurring value of attribute $a$.

The similarity values will be sorted in descending order. Only the similarity of the nearest neighbours will be used to compute the threshold by Eq. (3):

3 $$\mathrm{Threshold}=\left({\sum }_{i=1}^{k}\left(\frac{\left({\mathrm{Sim}}_{i} - {\mathrm{Sim}}_{i+1}\right)}{\left({\mathrm{Sim}}_{i}+ {\mathrm{Sim}}_{i+1}\right)/2}\right)\right) \times 0.5,$$

where $i=1, 2, \dots , k$ is the ordered list of similarity value in descending order and $k$ is the value of nearest neighbours.

The probability measure is computed according to the class label. The frequency counter is the key factor in determining the insertion of an object. It aims to keep track of the usage frequency of the nearest neighbours. The probability of each class label, $P\left({C}_{i}\right),$ is calculated based on the frequency counters for respective class label in the nearest neighbours, the total value of frequency counters in the nearest neighbours, and the total value of frequency counters for the class label. The formula to calculate the probability for each class is as follows:

4 $$P\left({C}_{i}\right)=\frac{\left(\sum_{i=1}^{k}{w}_{NN}\left({C}_{i}\right)\right)/\left(\sum {w}_{NN}\right)}{\left(\sum_{i=1}^{n}{w}_{{C}_{i}}\right)},$$

where ${C}_{i}$ is the class label, ${w}_{NN}$ is the number of frequency counters of is nearest neighbours, and ${w}_{{C}_{i}}$ is the number of frequency counters of the class label.

With the calculation of the percentage of difference in the nearest neighbours and the probability for each class, the insertion of an object takes place in two conditions as follows:

1. Insert an object if the percentage of the difference between the top two nearest neighbours is larger than the threshold, and the predicted class of the test object is different from the class label with the highest probability.
2. Insert an object if the percentage of the difference between the top two nearest neighbours is less than the threshold, and the predicted class of the test object is same with the class label with the highest probability.

In condition 1, if there is large difference between top two nearest neighbours and the predicted class is different with the class label with the highest probability, then the test object will be inserted to the training pool. The large difference indicates that the EEG signals have high inter-subject and low intra-subject variability [38]. Thus, it is believed that the prediction class label of the test object has high confidence to be classified correctly. On the other hand, the test object will be inserted to the training pool if the test object’s predicted class label is dissimilar from the class label with the highest probability. It is because the dissimilarity of the class labels showed the ambiguity. With the discernibility between the top two nearest neighbours, it is believed that the test object might be the new individual characteristic. Therefore, this test object will be inserted to the training pool and update the personal knowledge granules. Meanwhile, the insertion of object does not take place if the predicted class is same as the class label with the highest probability. It is because the existing knowledge granules are able to do the correct predictions. Therefore, the test object does not need to be included.

In condition 2, if there is small difference between top two nearest neighbours and the predicted class is same with the class label with the highest probability, then the test object will be inserted to the training pool. The small difference indicates that the top two nearest neighbours are similar to each other and showed the fuzziness between the top two nearest neighbours due to the nonstationary characteristics of the EEG signals. Besides that, there is a possibility where the top two nearest neighbours might come from different class labels. Thus, the comparison between the predicted class label and the class label with the highest probability should be carried out to validate the insertion decision. The test object will be inserted to the training pool if the test object's predicted class label is same as the class label with the highest probability. This gives higher confidence on correct prediction. In contrast, the test object will not be inserted if the predicted class label is different from the class label with the highest probability due to the ambiguity. The algorithm of the object insertion is shown in Algorithm 2 and the insertion of an object occurs if the test object fulfils either one of the conditions as described above.

### Deletion of object

In the proposed probability-based IncFRNN algorithm, the object deletion occurred when the window size threshold is defined. The main idea of window size threshold is to control the maximum number of training objects that can be stored in the training pool. Meanwhile, the frequency counter acts as a crucial role in the deletion of an object. The training object with the lowest count will be removed if the number of training objects reaches the window size threshold. The training object with the lower count indicates the object is less important and representative for the particular individual. Thus, the proposed probability-based IncFRNN algorithm eliminates the rarely used objects from the training pool. Furthermore, the proposed probability-based IncFRNN algorithm implements the First-In-First-Out (FIFO) strategy when the frequency counter for the training objects is the same. The algorithm of the deletion of an object is shown in Algorithm 2. The object deletion occurred if the window size threshold is larger than 0, and the number of training object is larger than the window size threshold.

## Materials and methods

**Fig. 1:**
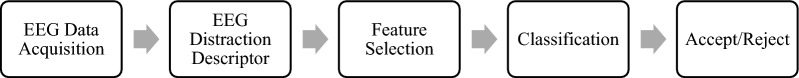
Brainprint authentication model

In this section, the process of brainprint authentication model is illustrated in Fig. 1. It comprises four components: data acquisition, EEG distraction descriptor, feature selection, and classification. In order to investigate the difference of neural responses towards the ambient noise, a new EEG experiment paradigm is developed.

### Data acquisition

A total of 45 healthy volunteer subjects (25 males and 20 females) were participated in developing the case study. The 45 subjects were selected based on predefined requirements, such as gender, health, age, and vision. The experimental subjects are selected based on three different age groups, such as 18–25 years old, 26–35 years old, and 36 years old and above. Each group is completed with 15 subjects. Ageing influences the distraction tolerant level in the auditory task [39]. All the volunteered subjects had a normal vision or corrected normal vision. Before conducting the experiment, a written consent form was signed by the volunteered subjects. The subject sat on a comfortable chair during the experimental process. It is to minimize the possible movements or artefacts during the recording session.

The electrodes were first attached on the subject’s scalp in order to begin the EEG data collection process. The head cap must be tightened to avoid connection loss, while maintaining comfortable to the subject. The electrode placements are based on the International 10–20 system. During the data collection, the reference and ground electrodes are right earlobe and right wrist, respectively. Meanwhile, the sample rate was set to 512 Hz. No filter was applied during EEG recording to avoid loss of information.

**Fig. 2:**
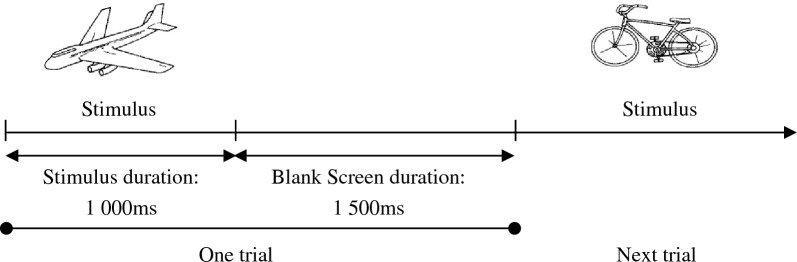
Visual stimulus presentation

The visual stimulus was displayed at the centre of a 15.6-inch computer monitor screen with the resolution of 1366 pixels × 768 pixels. The monitor was placed 1 m away from the subject’s eye level. The distance between the subject and the stimulus is normally ranged from 0.5 m to 1.5 m to reduce the subject’s attention loss due to eye fatigue effect on the near stimuli [40]. Before starting the experiment, the subjects were requested to choose a password image. When their password image appeared, the subjects were instructed to respond by clicking the mouse immediately. Each experimental session contains a total of 150 shuffled trials, where 60 trials consist of the pre-selected password image, and 90 trials are randomly selected images from the 260 images set, excluding the selected password image. Every trial had an Inter-Stimulus Interval (ISI) of 1.5 s. The image was remained on screen for 1 s and followed by 1.5 s of white-blank screen as shown in Fig. 2.

According to American Academy of Audiology [41], the level of noise is categorized into soft (30–40 dB), moderate (50–60 dB), and loud (70–80 dB). Thus, the experimental paradigm was carried out in three simulated conditions: (a) a quiet condition; (b) a low distraction condition; and (c) a high distraction condition. It is to mimic the different levels of distraction that happened in real-world situations. An audio clip with the regular office condition noise was played using the speakers, where the sound level was set to approximately 55 decibels (dB) to simulate the low distraction condition. In order to simulate a highly distractible environment, the same speakers were used to play an audio clip that contained irregular office environment sounds, such as printer printing, stamping, and phone ringing, at a sound level of 70 dB. Appropriate sound levels must be figured out to avoid the human ear being injured.

### EEG distraction descriptor

Filtering, segmentation, and artefact rejection were implemented to eliminate unwanted signals. A bandpass filter of Finite-duration Impulse Response (FIR) type, with the cut-off frequencies of 8 to 13 Hz and 13 to 30 Hz, was used to obtain the alpha and beta band signals. Next, the signals were segmented according to the trial. Then, followed by feature extraction, feature selection, and classification. The trials with the amplitude larger than 100 µV are considered body movements or other types of artefacts were discarded. Normal EEG signals range peaks from 0.5 to 100 µV [42]. Instead of using all the 21 electrodes, the EEG signals from five electrodes (T5, T6, O1, O2, and OZ) were selected for further analysis in this study to reduce the modelling complexity. They are selected based on their importance on visual and audio tasks. The occipital and the temporal electrodes are the dominant electrodes for visual and auditory, respectively [43].

#### Power spectral density (PSD)

Power spectral density (PSD) is defined as the signal strength distribution in the frequency domain [7]. An efficient method for converting EEG signals from the time domain to frequency domain is the Fast Fourier Transform (FFT). Due to the distinctive behaviours of the dynamic system, the signals contain the unique power concentrations in the frequency spectrum [44].

The PSD captures the correlation information between the measured signals from several electrode channels. PSD calculates the square of the absolute FFT data in each segment. The concavity of spectral distribution, the variance of spectral power, and the nondominant region of the power spectrum [45] were calculated as EEG features for further recognition purpose. The formula for Fourier Transform (FT) and PSD is shown in Eq. (5):

5 $$x\left(k\right)=\sum_{n=0}^{N-1}x\left(n\right){e}^{-j\left(\frac{2\pi }{N}\right)nk},$$

where $k=0, 1, 2, \dots , N-1$, $x\left(k\right)$ is the $k$th coefficient of the FT. The PSD of the EEG signal can be estimated directly, as shown in Eq. (6):

6 $${P}_{x}\left(k\right)=\frac{1}{N}{\left|x\left(k\right)\right|}^{2}.$$

The PSD coefficients of different frequency bands for the EEG signal are adopted as features.

#### Wavelet phase stability (WPS)

Wavelet phase stability (WPS) uses wavelet-based measure to quantify the phase information [46]. EEG signals composed of amplitude and phase information. Phase information in signal processing is more useful and stable than the amplitude information [8]. It is because the phase information takes into consideration of the nonstationary characteristics of the EEG signals. The time-resolved phase information assesses the quality and consistency of the response over the stimulus sequences. WPS has a range of 0 to 1, with 1 denoting the perfect phase stability. Lower phase stability is associated with lesser WPS values. The formula of WPS is defined as follows:

7 $${\Gamma }_{s,\tau }^{m}\left(\mathcal{F}\right)=\frac{1}{m}\left|\sum_{n=1}^{m}{e}^{larg\left(\left({\mathcal{W}}_{\psi }{f}_{m}\right)\left(s,\tau \right)\right)}\right|,$$

where ${\mathcal{W}}_{\psi }$ is wavelet transform of the signal, $m=1, \dots , M,$ and ${\Gamma }_{s,\tau }^{m}\left(\mathcal{F}\right)$ measures the mean of the degree of clustering of the angular distribution for certain $s$ and $\tau$ for $M$ trials.

#### Coherence

Coherence assesses the functional connectivity in the human cortex. EEG measures the neural dynamics to a functional brain state, which is determined by neuropathology, behaviour, and cognition [47]. It could provide information on how networks are formed and how different parts of the brain interact together. Furthermore, the coherence in the EEG signals provides an important approximation of functional interactions between the neural systems operating in each frequency band [47]. The coherence measures the degree of linear correlation between two signals. Coherence can reveal the correlation between two signals at different frequencies. The analysis of EEG data has been used in numerous clinical and cognitive situations. Coherence measures the degree of synchronization between the frequency components of two signals and provides the estimation of functional connectivity of the brain [48].

The magnitude range of the coherence is estimated between 0 and 1, which quantifies the correspondence between $x$ and $y$ at each frequency. The value of 0 in the coherence function indicates the independence between two signals and vice versa. The higher the value of the coherence, the higher the linear dependence between the two signals. The expression of coherence given as follows:

8 $${C}_{xy}\left(f\right)= \frac{{\left|{P}_{xy}\left(f\right)\right|}^{2}}{{P}_{xx}\left(f\right){P}_{yy}\left(f\right)},$$

where ${C}_{xy}\left(f\right)$ is a function of the power spectral density $\left({P}_{xx}\mathrm{ and }{P}_{yy}\right)$ of $x$ and $y$ and the cross-power spectral density $\left({P}_{xy}\right)$ of $x$ and $y$.

### Feature selection

Feature selection is a process to reduce the dimension of the feature vectors without jeopardized the classification performance. Correlation-based Feature Selection (CFS) is a useful feature selection technique to diminish the dimension of the input data without influencing the classification performance. CFS has been widely used in supervised learning [49] and time series data [50]. Kabir et al. [51] investigated the feature selection techniques and the results showed that CFS is performed better than minimum redundancy and maximum relevance (mRMR), and multi-subspace randomization and collaboration-based unsupervised feature selection (SRCFS). A representative feature subset should contain a high correlation between the features and the target class. The CFS algorithm chooses the best inter-correlated feature subset according to the correlation-based heuristic merit [52]. The heuristic merit is calculated as

9 $${\mathrm{Merit}}_{S}=\frac{k\overline{{r }_{cf}}}{\sqrt{k+k\left(k-1\right)\overline{{r }_{ff}}}},$$

where ${\mathrm{Merit}}_{S}$ is the heuristic “$\mathrm{merit}$” of a feature subset $S$ containing $k$ features, $\overline{{r }_{cf}}$ is the mean feature-class correlation $\left(f\in S\right)$, and $\overline{{r }_{ff}}$ is the average feature of the inter-correlation. The heuristic handles the irrelevant features as these features will be the poor predictors of the class. It discriminates against the redundant attributes as these features will be highly correlated with one or more of the other features.

Only 12 out of 210 features were selected for the brainprint authentication modelling in this study. Among the 12 features, only two features were selected from coherence measure, which are the coherence of O1 and OZ channels and the coherence of OZ and O2. Meanwhile, all the features from PSD and WPS of T5, T6, O1, OZ, and O2 were selected. This has proven that the PSD is able to study the degree of human attention that affected by the sound levels in EEG signals [53]. Besides, the WPS is also significant to neural correlation of attention to the auditory signals [46]. Since brainprint authentication is a binary class problem, the output class will be either client or impostor instead of the number of subjects. A 10 folds cross-validation will be used on training and testing data to avoid biased performance during the measurement. In an incremental learning framework, the initial training size can be reduced as the knowledge is learned from time to time [54]. Hence, only about 10% of the dataset was used as the training set, while the remaining 90% was used as the testing set. Incremental Fuzzy-Rough Nearest Neighbour (IncFRNN) technique was used to perform classification to evaluate the authentication performance.

### Classification

EEG signals classification is a tricky task as the EEG signals changing from time to time [55]. EEG signals have high dimensionality and very low signal-to-noise ratio. In addition, the EEG signals may differ between the acquisition sessions even though the subjects perform the similar tasks when respond to the same stimuli. Hence, the classifier with the capability of incremental learning is necessary to include the new individual EEG signals characteristics in enhancing the classification performance for brainprint authentication.

The proposed probability-based incremental update strategy is benchmarked with the ground truth (actual class) incremental update strategy [5]. Besides, the proposed technique also benchmarked with the First-In-First-Out (FIFO) incremental update strategy in K-Nearest Neighbour (KNN). Both IncFRNN and KNN are instance-based learning and can be found in the Waikato Environment for Knowledge Analysis (WEKA). Incremental K-Nearest Neighbour is available in WEKA and it is known as Instance-based Learning with parameter $k$ (IBk) classifier.

KNN is a common classification technique, and it is an example of instance-based learning technique. KNN algorithm used the Euclidean distance to compute the distance between training and testing data. The class of the test object will be predicted once the training object’s closest distance has been located. The value of $k$ represents the space of the neighbourhood around the test object. However, the value of $k$ is more suitable if it is an odd value [56]. The results obtained in research work [57] indicated that the accuracy of the number of $k$-value with odd value is better than the number of $k$-value in even number. Nevertheless, the classification efficiency will increase if the value of $k$ is set appropriately. A range of parameter $k$ from 1 to 100 was tested for EEG-based person identification [58]. An optimal value of $k=5$ is obtained and achieved 100% in accuracy. By default, the KNN uses one nearest neighbour $\left(k=1\right),$ but the number can be defined manually. The time required for classifying the test object is increased linearly with the number of training objects. Therefore, it may occasionally be required to limit the size of the training pool. It can be done by setting the window size threshold [59]. The window size determines the maximum number of objects allowed in the training pool. The removal of older objects occurs when the number of training objects is larger than the value of window size.

Some parameters are required to set for performing the classification in Knowledge Flow WEKA. The number of $k$ is the number of nearest neighbours which determine the coming object compares itself to those nearest neighbours. The value of $k$ is set to 5 [58] for the proposed probability-based IncFRNN, IncFRNN, and KNN classifier. On the other hand, the window size threshold is set for the proposed IncFRNN and IBk classifier to limit the size of training pool. By default, the value of window size is 0, meaning that there is no restriction on the number of training objects. A preliminary study was carried out to evaluate the impact of window size threshold. The window size threshold is tested with a 10% increment, which is equivalent to 675 objects. Initially, the dataset was split into 10% for training objects and 90% for testing objects. Thus, the window size threshold analysis is started at 20%, which is equivalent to 1350 objects. The overall classification performance when using 60% of training data is similar to 70% of training data. Thus, the window size threshold is set to 60%, which is equivalent to 4050, because it is enough to achieve good classification performance. The lower the window size threshold, the lesser the complexity for the classification.

### Performance measurement and validation test

Brainprint authentication model only produces two classes, which are yes or no. Thus, area under Receiver Operating Characteristic (ROC) curve (AUC), recall, and accuracy were used to analyse the experimental results. The AUC is normally interpreted in a graph and used the simple trapezoidal integration to calculate the area under the curve. It is used to make a balance between the true-positive rate and false-positive rate. On the other hand, the accuracy assesses the efficiency of the classifier by its percentage of true predictions. In binary classification, the recall denotes the number of correct predicted positive examples divided by the total number of positive examples in the dataset. The recall is also similar to True-Positive Rate (TPR). Therefore, the higher the value of recall, the better the classification performance. An Anderson–Darling test was used to examine the normality distribution of the results prior to the validation test.

Paired sample t-test is one of the validation tests that compares the mean from various dataset. The goal is to examine whether there is significant difference between the paired samples. Eq. (10) shows the comparison of the difference of the mean between the samples $\left(\overline{D }\right)$ to the difference that we would expect to find between population means $\left({\mu }_{D}\right)$ and then takes into the standard error of the differences $\left({S}_{D}/\sqrt{N}\right)$. A significant value in paired sample t-test shows the two-population means are statistically different, if and only if the *p*-value is smaller than or equal to 0.05. On the other hand, there is not statistically significant different between the two samples when the *p*-value is larger than 0.05. In this study, the model with higher authentication results is significantly performed better if and only if the *p*-value is larger than 0.05.

10 $$t=\frac{\overline{D }-{\mu }_{D}}{{S}_{D}/\sqrt{N}}.$$

## Experimental results

In this section, the experimental results are validated from two perspectives, which are the comparison of incremental update strategies and the comparison of incremental learning models. For the comparison of incremental update strategy, both probability-based and actual class incremental update strategies are implemented in IncFRNN technique. Meanwhile, the probability-based IncFRNN and IBk techniques are selected to evaluate the classification performance of incremental learning models. The classification performance was evaluated based on the AUC, accuracy, recall, precision, and F-Measure. Furthermore, a validation test with a 95% confidence level was carried out to test the significant difference for each comparison.

**Fig. 3:**
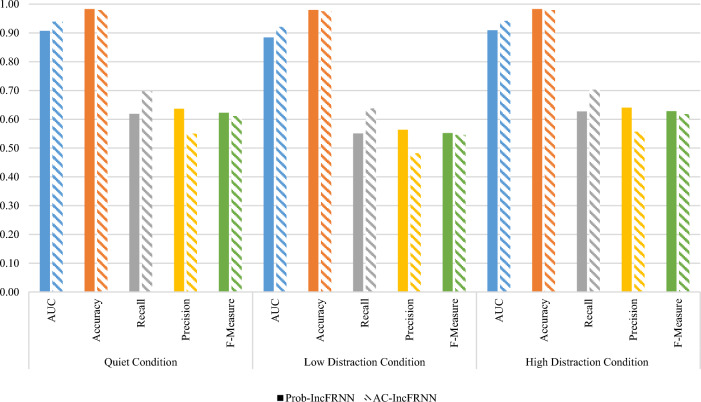
Comparison of probability-based and actual class incremental update strategies

Figure 3 shows the comparison of the experimental results between the probability-based and actual class incremental update strategies in IncFRNN technique. The authentication performances were tested in three different environmental conditions. The AUC and recall of actual class incremental update strategy are higher than the probability-based incremental update strategy. The AUC of the actual class incremental update strategy achieved 0.9387, 0.9205, and 0.9422 for the EEG recorded in the quiet, low distraction, and high distraction conditions, respectively. However, the AUC of the proposed probability-based incremental update strategy obtained 0.9074, 0.8842, and 0.9096 for each dataset. On the other hand, the recall of probability-based incremental update strategy is slightly lower than the actual class incremental update strategy, with the difference of 0.0774, 0.0860, and 0.0750 for each dataset. The validation tests showed significant difference for the comparison of the probability-based and actual class incremental update strategies in terms of AUC and recall. Since the AUC and recall of the actual class incremental update strategy are higher than the probability-based incremental update strategy, it can be concluded that the actual class incremental update strategy is performed better than the probability-based incremental update strategy in all the three environment conditions.

In contrast, the accuracy, precision, and *F*-Measure of the probability-based incremental update strategy are slightly higher than the actual class incremental update strategy. In terms of accuracy, the probability-based incremental update strategy gained 0.9833, 0.9802, and 0.9830, while the actual class incremental update strategy obtained 0.9796, 0.9755, and 0.9797 for each dataset. Meanwhile, the difference of precision between two incremental update strategies are 0.0868, 0.0824, and 0.0843 for quiet, low distraction, and high distraction, respectively. The validation tests showed the accuracy and precision of probability-based incremental update strategy are significantly better than the actual class incremental update strategy. On the other hand, both probability-based and actual class incremental update strategies achieved the highest F-Measure in high distraction condition, which are 0.6285 and 0.6171, respectively. The F-Measure in quiet condition is slightly lower, with the difference 0.0055 and 0.0060 only for probability-based and actual class incremental update strategies. The validation tests showed the F-Measure of probability-based incremental update strategy are significantly higher than the F-Measure of actual class incremental update strategy in both quiet and high distraction conditions. Nevertheless, the *p*-value of the result pairs in low distraction condition is 0.072, which is greater than 0.05. Thus, it is no significant difference for the comparison of probability-based and actual class incremental update strategies in low distraction condition. Moreover, the low distraction condition yielded the lowest F-Measure for both probability-based and actual class incremental update strategies. The higher value of F-Measure in probability-based incremental update strategy was contributed by precision.

In the overall comparison between the classification performance of probability-based incremental update strategy and the actual class incremental update strategy, it can be concluded that the actual class incremental update strategy works better than the probability-based incremental update strategy. In other words, the probability-based incremental update strategy is not as good as the actual class incremental update strategy. The actual class incremental update strategy in the IncFRNN algorithm represents the ground truth situation. Thus, it is reasonable that the classification performance of the actual class incremental update strategy is better than the probability-based incremental update strategy.

**Fig. 4:**
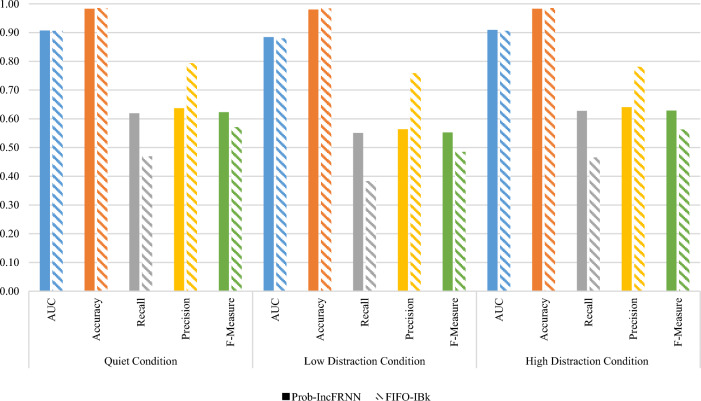
Comparison of probability-based IncFRNN and IBk techniques

Apart from comparing the actual class incremental strategy, the probability-based incremental update strategy in IncFRNN technique also benchmarked with the First-In-First-Out (FIFO) incremental update strategy that found in the K-Nearest Neighbour (KNN) technique. KNN is an incremental learning technique and is known as Instance-Based with parameter *k* (IBk) in WEKA. Figure 4 shows the experimental results and validation tests to compare the probability-based IncFRNN and IBk techniques.

From the AUC as shown in Fig. 4, the AUC of probability-based IncFRNN technique is slightly higher than IBk technique, with the difference of 0.0018 only. The *p*-value of this comparison is 0.422, which is greater 0.05. Therefore, there is no significant difference between the comparison techniques. This result showed that both probability-based IncFRNN and IBk are performed well in the quiet condition. The authentication result in the high distraction condition is similar to quiet condition. The AUC of probability-based IncFRNN technique achieved 0.9096, which is 0.0033 higher than IBk technique. With the small difference between both techniques, the validation test showed no significant difference. In contrast, the validation test shows significantly different for the authentication results in low distraction condition, with the *p*-value being 0.0027. Thus, it is proven that the probability-based IncFRNN technique performs better than the IBk technique in low distraction condition.

From this assessment, it can be concluded that the probability-based IncFRNN technique is more suitable to be used for the brainprint authentication model, although the classification performance in AUC, recall, and F-Measure was contradicted with the precision and accuracy. F-Measure, recall, and precision were the preferred performance measures compared to accuracy and AUC. It is because of the imbalanced class distribution problem with the ratio of 1 to 45 in this brainprint authentication model. The higher accuracy of IBk technique was majority contributed by true-positive rate (correct prediction as impostor class), and very minor was contributed by the true-positive rate (correct prediction as client class). The accuracy was biased towards the class with a large number of training objects, the impostor class in this experiment. It is not favourable and should be avoided in an authentication model evaluation because the number of impostors increases with the total number of users in the system.

**Fig. 5:**
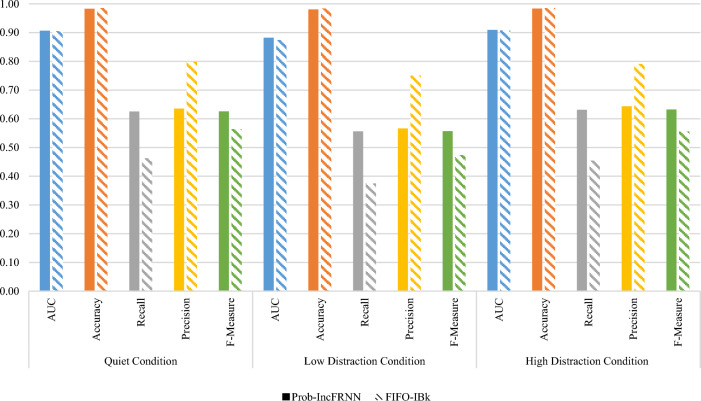
Comparison of probability-based IncFRNN and IBk techniques with window size threshold

Window size threshold is required if the user would like to control the size of the training pool. The size of the training pool is getting increases if the test objects are continuously inserted into the training pool. It will lead to a large training pool and increase computational complexity. Thus, it is vital to retain the useful and unique characteristics in the training pool for describing an individual. A comparison of probability-based incremental update strategy in the IncFRNN technique and the First-In-First-Out (FIFO) incremental update strategy in the IBk technique was tested with window size threshold. The probability-based incremental update strategy deletes the object based on frequency counter, while the FIFO incremental update strategy deletes the oldest object. The classification performance and validation test are shown in Fig. 5.

In terms of AUC, the validation tests showed not significantly different for the EEG signals recorded in the quiet and high distraction conditions, with the *p*-value greater than 0.05. In the quiet condition, the AUC is recorded at 0.9066 when using the probability-based IncFRNN technique and 0.9045 when using the IBk technique. In the high distraction condition, the probability-based IncFRNN technique gained 0.9093, while the IBk technique obtained 0.9069 in terms of AUC. In contrast, the *p*-value for comparing probability-based IncFRNN and IBk techniques in low distraction condition is 0.007, which is less than 0.05. Thus, there are significantly different. The AUC acquired from the probability-based IncFRNN technique, 0.8826, is higher than the IBk technique, 0.8736. Hence, it can be concluded that the probability-based IncFRNN technique is performed better than the IBk technique. On the contrary, all the validation tests in terms of accuracy showed significantly different, with the *p*-value equals to 0.000. The IBk technique gained a higher value as compared to probability-based IncFRNN technique. The higher accuracy obtained by the IBk technique is due to the true negative rate. In this brainprint authentication scenario, a large amount of the data are contributed by the impostor class. Therefore, the classification performance is further analysed using recall, precision, and F-Measure.

In terms of recall, the probability-based IncFRNN technique gained 0.6253, 0.5564, and 0.6312, which are 0.1627, 0.1823, and 0.1770 higher than the IBk technique in quiet, low distraction, and high distraction conditions, respectively. The recall of the IBk technique is around 0.4 only for both quiet and high distraction conditions, which are very poor classification performance. The *p*-value of this comparison is 0.000, which indicates that the probability-based IncFRNN technique works better than the IBk technique. Based on the precision in Fig. 5, the IBk technique achieved the higher values as compared to the probability-based IncFRNN technique, with the *p*-value equal to 0.00 for all the datasets. The IBk technique gained 0.7970, 0.7501, and 0.7904 for the quiet, low, and high distraction conditions, respectively. Meanwhile, the precision of the probability-based IncFRNN technique was recorded at 0.6356, 0.5662, and 0.6437 for each dataset. The high precision in IBk techniques might because of the imbalanced class distribution. In biometric authentication model, the majority class label is contributed by the impostor. The IBk technique biased the data with majority class label. Indirectly, it will decrease the misclassification rate on the impostor class and the number of false positives in client class.

In terms of F-Measure, the probability-based IncFRNN technique yielded higher classification results as compared to the IBk technique. The F-Measure of the probability-based IncFRNN technique were 0.6257, 0.5571, and 0.6322 for the quiet, low distraction, and high distraction conditions, respectively. On the other hand, the F-Measure of IBk technique were 0.5642, 0.4732, and 0.5551 only for each dataset. It is considered a poor classification performance. This experiment proved the deletion process in probability-based IncFRNN technique is more suitable for brainprint authentication modelling.

## Discussions

As been explained in the above sections, the experimental results and validation tests have shown that the actual class incremental update strategy in the IncFRNN technique was performed better than the proposed probability-based incremental update strategy in the IncFRNN technique. In other words, the object insertion based on the probability method does not perform as good as the actual class. The use of actual class in the incremental update strategy of IncFRNN algorithm acts as the ground truth situation. Therefore, it is reasonable on the classification performance of actual class IncFRNN is better than the probability-based IncFRNN techniques. However, it is almost impossible to get the actual class label in brainprint authentication applications. Thus, incrementally updating the training pool based on the probability method is proposed to solve the issue of using the actual class label. In order to evaluate the probability-based incremental update strategy in IncFRNN technique, it is benchmarked with the First-In-First-Out (FIFO) incremental update strategy, which is available in the IBk technique.

The proposed probability-based IncFRNN technique is worked better than its benchmark strategy, the IBk technique for both with and without defining the window size threshold from the experimental results and validation tests. The deletion of an object will only be tested if the window size threshold is defined. The probability-based IncFRNN technique deletes the object based on the frequency counter, while the IBk technique deletes the object based on the queue to control the size of the training pool. In other words, the FIFO incremental update strategy deletes the oldest object in the training pool without the consideration of the class label. It will be a big issue for the imbalanced class dataset because the FIFO incremental update strategy may delete all the training data with minority class. The window size threshold analysis was performed to determine the ideal size of training pool. It is because the classification performance will be degraded if the size of the training pool is small and unable to store the individual representative characteristics for the brainprint authentication.

With the object insertion based on the probability method and object deletion based on the frequency counter, the training pool will be updated with the new objects to enhance the classification performance. Simultaneously, the old and obsolete objects will be removed from the training pool to prevent the size of training pool from growing. From the perspective of brainprint authentication, the new individual characteristics of EEG signals will be added into the knowledge granules by inserting the object, while the old and rarely used individual EEG signal characteristics will be removed by deleting the object. It is because the characteristics are less meaningful to be used as the identity for the particular individual due to the changes in EEG signals over time.

## Conclusion

The new version of the IncFRNN algorithm comes with the probability-based incremental update strategy. Instead of using the actual class label to update the training pool, the probability-based incremental update strategy is proposed to calculate the probability the reliability of each class label. The experimental results showed that the probability-based incremental update strategy is not performed as good as the actual class incremental update strategy. It is because the actual class label represents the ground truth. On the other hand, the experimental results have proven that the probability-based incremental update strategy in IncFRNN technique is worked better than the First-In-First-Out (FIFO) incremental update strategy in IBk technique for both the object insertion and object deletion. Nevertheless, the object deletion is an optional process. The deletion of an object takes place if the window size threshold is defined. With the nature of EEG signals, the probability-based IncFRNN algorithm is more suitable for the brainprint authentication model. The probability-based IncFRNN technique stores the representative objects and eliminates the old and rarely used objects. By doing this, the current individual characteristics have been preserved, and the past individual characteristics are abolished. However, the EEG distraction descriptor may vary due to intersession variability. Future work may focus on the intersession variability to enhance the robustness of the brainprint authentication model.

## Data Availability

Not applicable.
